# Effect of Silver Nanoparticles on the Microstructure, Non-Isothermal Crystallization Behavior and Antibacterial Activity of Polyoxymethylene

**DOI:** 10.3390/polym12020424

**Published:** 2020-02-12

**Authors:** Yicheng Zeng, Yang Liu, Lumin Wang, Hongliang Huang, Xun Zhang, Yongli Liu, Minghua Min, Ying Li

**Affiliations:** 1 Key Laboratory of Oceanic and Polar Fisheries, Ministry of Agriculture and Rural Affairs, East China Sea Fisheries Research Institute, Chinese Academy of Fishery Sciences, Shanghai 200090, China; yicheng_1026@163.com (Y.Z.); lmwang300@126.com (L.W.); ecshhl@163.com (H.H.); zhangxun007@hotmail.com (X.Z.); 1981-lyl@163.com (Y.L.); 2 College of Materials Science and Engineering, University of Shanghai for Science and Technology, Shanghai 200093, China; 3 Hunan Xinhai Co., Ltd., Hunan 413100, China; 13917177635@139.com

**Keywords:** POM, Ag nanoparticles, POM/Ag nanocomposites, non-isothermal crystallization kinetics, heterogeneous nuclei, antibacterial activity

## Abstract

Silver (Ag) nanoparticles were synthesized by a facile route in the presence of oleic acid and n-propylamine. It was shown that the average primary size of the as-synthesized Ag nanoparticles was approximately 10 nm and the surface of as-synthesized Ag nanoparticles was capped with monolayer surfactants with the content of 19.6%. Based on as-synthesized Ag nanoparticles, polyoxymethylene (POM)/Ag nanocomposites were prepared. The influence of Ag nanoparticles on non-isothermal crystallization behavior of POM was investigated by differential scanning calorimetry (DSC). The Jeziorny, Jeziorny-modified Avrami, Ozawa, Liu and Mo, Ziabicki and Kissinger models were applied to analyze the non-isothermal melt crystallization data of POM/Ag nanocomposites. Results of half time (*t*_1/2_), crystallization rate parameter (CRP), crystallization rate function (*K*(*T*)), kinetic parameter (*F*(*T*)), the kinetic crystallizability at unit cooling rate (*G_Z_*) and the crystallization activation energy (∆*E*) were determined. Small amounts of Ag nanoparticles dispersed into POM matrix were shown to act as heterogeneous nuclei, which could enhance the crystallization rate of POM, increase the number of POM spherulites and reduce POM spherulites size. However, the higher loading of Ag nanoparticles were easily aggregated, which restrained POM crystallization to some degree. Furthermore, the POM/Ag nanocomposites showed robust antibacterial activity against *Escherichia coli* and *Staphylococcus aureus*.

## 1. Introduction

Ag nanoparticles have received significant attention owing to their unique volume effect and quantum size as well as their high conductivity, excellent catalytic performance and broad spectrum of antimicrobial activities [1,2]. Ag nanoparticles have been extensively used in nonaqueous conductive ink [3], hydrogen sorption and storage [4], antibacterial materials [5], bone tissue regeneration and wound repair [6,7], etc. Thus, polymer/Ag nanocomposites have widely been applied in a variety of areas, such as the microelectronics, optoelectronics, magnetic materials, catalysis, chemical sensors and biology [8,9,10]. One of the interesting studies was that of the effect of polyethylene (PE)/Ag nanocomposites on Ag ion release and antimicrobial properties by Zapata et al. [11]. It was found that PE/Ag nanocomposites of higher Ag nanoparticle concentrations (5 wt%) showed the highest Ag ion release and reached 99.99% efficacy against the bacteria after 24 h. Shi et al. [12] investigated the influences of Ag content and crystallization temperature on the crystallization behavior and crystalline structure of polypropylene (PP)/Ag nanocomposites. They found that the PP nanocomposites with Ag nanoparticles had higher crystallization rate constant and the Avrami exponent than pure PP, indicating that the Ag nanoparticles acted as heterogeneous nucleating sites and increased the crystallization rate of PP. Liu et al. [13] designed and prepared novel hybrid films which consisted of gelatin-g-poly(methyl methacrylate) (PMMA)/Ag with ordered nanopores. The antibacterial activities of hybrid films against Escherichia coli and Staphylococcus aureus were found with the disc diffusion method and colony count assays. The results showed that the gelatin-g-PMMA/Ag nanocomposites of 50 μg/mL concentration had clear and lasting antibacterial activity. A more sophisticated ex situ preparation method was developed to prepare polyurethane (PU)/Ag nanocomposites by Triebel et al. [14]. They investigated the Ag ion release and the antimicrobial efficacy of PU/Ag nanocomposites. The results demonstrated that the PU/Ag nanocomposites with 0.07 wt% Ag nanoparticles exhibited a high release of Ag ions and good antimicrobial properties.

Polyoxymethylene (POM) is an engineering semicrystalline thermoplastic with excellent surface lubrication, outstanding antifatigue performance, high electrical insulation and good chemical and weathering resistance [15,16]. POM exhibited higher crystallinity than other semicrystalline polymers, which led to gap-sensitivity and low impact toughness of POM and limited the application of POM in industry [17,18]. Therefore, many studies have reported on the effect of nucleating agents on the crystallization performance of POM [19,20]. Czarnecka-Komorowska et al. [21] investigated the effects of octakis((3-glycidoxypropyl) dimethylsiloxy) octasilsesquioxane (GPOSS) on the crystallinity, crystal structure, morphology and mechanical properties of POM. They demonstrated that the addition of GPOSS reduced the spherulite size of POM and improved the crystallization growth rate and the degree of crystallinity of POM due to the nucleation effect of GPOSS in polymers. Zhao et al. [22] studied the effect of multiwalled carbon nanotubes (MWCNTs) on the crystallization behavior of POM. They pointed out that MWCNTs reduced the induction time of crystallization and improved the crystal growth rate and crystallization temperature of POM.

In this work, we successfully synthesized Ag nanoparticles with a monolayer of surfactants which was composed of oleic acid and n-propylamine. Then, we prepared POM/Ag nanocomposites by a simple melt compounding route. The effect of Ag nanoparticles on the microstructure and non-isothermal crystallization behavior of POM/Ag nanocomposites were investigated by scanning electron microscopy (SEM) and differential scanning calorimetry (DSC), respectively. In addition, the spherulite morphology of POM/Ag nanocomposites was observed by polarized optical microscopy. Furthermore, the antibacterial characteristics of POM/Ag nanocomposites were also studied.

## 2. Experimental

### 2.1. Materials

Polyoxymethylene (POM) was obtained by Yunan Yuntianhua Co., Ltd., Shanghai, China. Silver nitric (99.8%), oleic acid, ethanol, n-propylamine, ascorbic acid (99.7%) and n-heptane were purchased from Sinopharm Chemical Reagent Co., Ltd., Shanghai, China. Agar powder, tryptone and beef extract were purchased from Aladdin Bio-Chem Technology Co., Ltd., Shanghai, China. All the reagents employed in this work were analytical grade and were used as received.

### 2.2. Synthesis of Ag Nanoparticles

**Figure 1:**
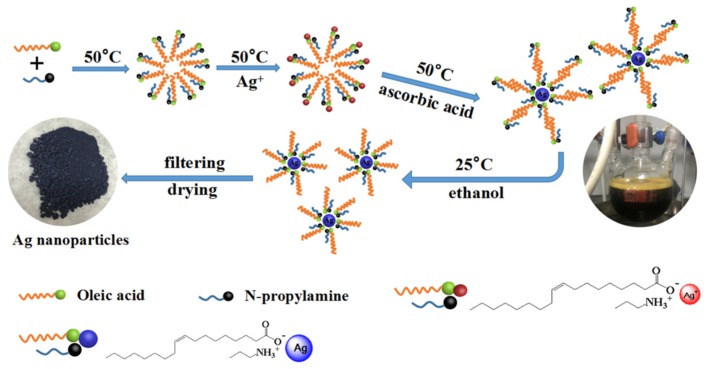
Synthesis procedure of Ag nanoparticles.

The AgNO_3_ (3.397 g of AgNO_3_ dissolved in 200 mL of deionized water), oleic acid (25 mL) and n-propylamine (75 mL) were stirred at a high speed in a 500 mL three-necked flask at 50 °C for 1 h [23]. Then, ascorbic acid solution (7.045 g of ascorbic acid dissolved in 50 mL of deionized water) was added. The mixture was held at 50 °C and stirred for 3 h, and a dark brown organic Ag nanoparticle colloidal solution was obtained. After that, ethanol (500 mL) was added into the solution to precipitate the crude Ag nanoparticles. Finally, the precipitate was isolated by filtration, washed twice with acetone and vacuum-dried at 50 °C for 48 h to afford the final products in the form of blue powders. Synthesis procedure of Ag nanoparticles is shown in Figure 1.

### 2.3. Preparation of POM/Ag Nanocomposites

POM and the Ag nanoparticles were vacuum-dried at 80 °C for 24 h before use. Then POM/Ag nanocomposites were prepared by melt blending method using a torque rheometer. The contents of Ag nanoparticles were fixed at 0.1, 0.5, 1 and 2 wt%. The roller speed was 60 rpm and the process temperature was maintained at 180 °C. The mixing process lasted for 10 min to achieve homogeneous blending and was used for preparation of the POM/Ag nanocomposites.

### 2.4. Fourier Transform Infrared Spectroscopy (FTIR)

FTIR measurements were performed on an infrared spectrometer (Spectrum 100, PerkinElmer, Shanghai, China). All spectra were measured within the 500–4000 cm^−1^ region with KBr pellets.

### 2.5. Transmission Electron Microscopy (TEM)

The morphology of the Ag nanoparticles was investigated by TEM (Tecnai G2 F30, FEI, Hillsboro, OR, USA). All TEM samples were prepared by placing a drop of Ag nanoparticles in n-heptane onto an amorphous carbon-coated copper grid. The size distribution of the Ag nanoparticles was analyzed using Image J software.

### 2.6. Scanning Electron Microscopy (SEM)

Morphological and microstructural features of POM/Ag nanocomposites were investigated by SEM (Quanta 450, FEI, Hillsboro, OR, USA), and the cross-sectioned samples were prepared by fracturing the POM/Ag nanocomposites in liquid nitrogen. The Ag nanoparticles in the POM/Ag nanocomposites were also characterized by energy dispersive X-ray spectroscopy (EDS) analysis on the sample images.

### 2.7. Thermogravimetric Analysis (TGA)

Thermal stability experiments were performed by using a TG analyzer (Pyris I, PerkinElmer, Shanghai, China) under a nitrogen environment at a heating rate of 10 °C/min from 25 to 650 °C.

### 2.8. X-ray Diffraction (XRD)

The Ag nanoparticles were examined by using an X-ray diffractometer (D8 Advance, Bruker, Beijing, China) with Cu-Kα radiation. The diffractograms were scanned in a 2θ range from 20° to 90° at a scanning speed of 6°/min. The generator was operated at 40 KV and 200 mA.

### 2.9. Differential Scanning Calorimetry (DSC)

The DSC measurements of POM/Ag nanocomposites were performed on a DSC 204F1 (Netzsch, Selb, Germany). Samples weighing about 5–8 mg in an aluminum crucible were heated from 30 to 200 °C with the heating rate of 10 °C/min and kept at 200 °C for 5 min to remove the thermal history, then cooled from 200 to 30 °C with the cooling rates of 5, 10, 20, 30 and 40 °C/min.

Degree of crystallinity (*X_C_*) was calculated by the Equation (1) to examine the crystallinity change caused by the addition of the Ag nanoparticles [24]:

(1) $$X_{C}\left(\%\right)=\frac{\Delta H_{m}}{\left(1-\alpha \right)\Delta H_{m}^{o}}\times 100$$

where ∆*H_m_* is the enthalpy of melting in the second heating scan of the samples (J/g), ∆$H_{m}^{o}$ is the enthalpy value of melting of a 100% crystalline form of matrix polymer and α is the weight fraction of Ag nanoparticles. The ∆$H_{m}^{o}$ value of POM was 326 J/g [25].

### 2.10. Polarized Optical Microscopy

Polarized optical microscopy was performed to observe the crystalline morphology of POM/Ag nanocomposites on a polarizing microscope (Axio Imager A2, Zeiss, Shanghai, China) equipped with a digital camera (Axiocam Mrc 5, Zeiss, Shanghai, China). Each sample was sandwiched between two thin glass sides, kept at 180 °C for 5 min to remove any thermal history on the hot stage, then cooled to 25 °C at a rate of 10 °C/min. The photographs were then taken.

### 2.11. Antibacterial Tests

The paper-disk diffusion method was employed to assay the antibacterial activities of the POM/Ag nanocomposites. *Escherichia coli* (ATCC: 9739) and *Staphylococcus aureus* (ATCC: 6538) were selected as bacterial models. The medium used for growing and maintaining the bacterial liquid cultures was Luria-Bertani (LB) medium, and a solid medium was obtained by adding agar into the liquid medium. The POM/Ag nanocomposites were uniformly placed onto the filter paper discs (2 cm in diameter) and dispersed on the agar plates with either *E. coli* (5 mL, 10^5^ colony forming units (CFU) per mL) or *S. aureus* (5 mL, 10^5^ CFU per mL). The diameters of the inhibition zones were measured after incubation at 37 °C for 24 h.

In order to further elucidate the antibacterial efficiency of the POM/Ag nanocomposites and the inhibition rates of the POM/Ag nanocomposites with respect to the different Ag nanoparticle quantities, the bacterial densities of activated *E. coli* and *S. aureus* were first diluted from 10^8^ CFU/mL to 10^6^ CFU/mL with broth. Then, the POM/Ag nanocomposites were mixed with 50 mL of bacterial suspension and incubated at 37 °C for 24 h. UV absorbance (OD) of the strain broth at 630 nm was measured and used for the calculation of inhibition rate. The inhibition rate was calculated by the Equation (2) [26], where *A*_0_ corresponds to the OD value of the culture broth before culture; *A_t_* represents the OD value of the test samples and *A_con_* is the OD value of the mixed solution of broth and saline after incubation for 24 h:

(2) $$\mathrm{Inhibition}\;\mathrm{rate}\;(\%)=100-100((A_{t}-A_{0})/(A_{\mathrm{con}}-A_{0}))$$

## 3. Results and Discussion

### 3.1. Characterization of Ag Nanoparticles

**Figure 2:**
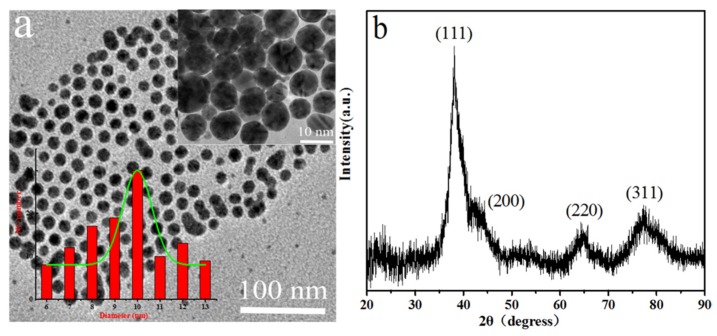
(**a**) TEM images of the as-synthesized Ag nanoparticles and the corresponding size distribution of as-synthesized Ag nanoparticles (inset); (**b**) XRD pattern of as-synthesized Ag nanoparticles.

**Figure 3:**
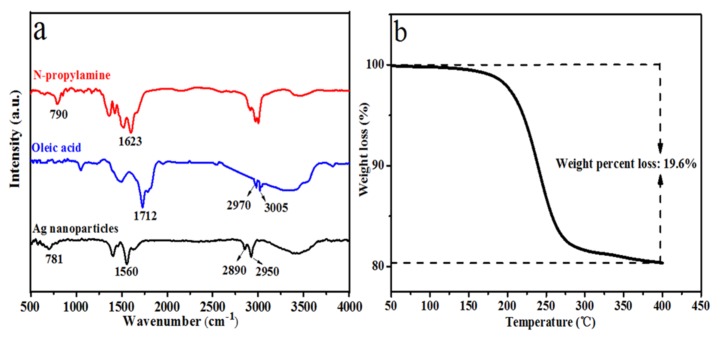
(**a**) FTIR spectra of n-propylamine, oleic acid and the Ag nanoparticles; (**b**) TGA curve of as-synthesized Ag nanoparticles.

Figure 2a shows the TEM images of Ag nanoparticles. It was clear that each Ag nanoparticle was well separated from the neighboring ones, indicating that the Ag nanoparticles were well surface-passivated by the stabilization action of the n-propylamine and oleic acid. Again, it could be found that the average size of Ag nanoparticles was mainly 10 nm, with a narrow size distribution from the corresponding size distribution of Ag nanoparticles (Figure 2a inset). In addition, the XRD pattern of the Ag nanoparticles is shown in Figure 2b. It was in good agreement with the literature value for the crystal structure of Ag nanoparticles [27]. It was believed that the prominent peaks of 2θ values at 38.1°, 42.1°, 64.5° and 77.5° represented the 111, 200, 220 and 311 Bragg’s reflections of the face-centered cubic crystal structure of Ag. Furthermore, the FTIR spectra of oleic acid, n-propylamine and Ag nanoparticles are shown in Figure 3a. Two absorption peaks were observed at 790 cm^−1^ and 1623 cm^−1^ on the spectrum of the n-propylamine, which were attributed to the N–H vibration peak. Meanwhile, in the FTIR spectrum of pure oleic acid, the broad peak of 2500–3600 cm^−1^ belonged to the O-H stretching vibration peak in the carboxyl group, and 2970 cm^−1^ and 3005 cm^−1^ corresponded to the C–H stretching vibration peak beside the double bond and the C–H stretching vibration peak in the CH_2_, respectively [28,29]. As for the spectrum of Ag nanoparticles, this showed the characteristic adsorption peaks at 2890 cm^−1^ and 2950 cm^−1^, which were attributed to C–H stretching vibration band of oleic acid. This indicated that the oleic acid molecules adsorbed on the surface of Ag nanoparticles changed the solid field of the Ag nanoparticles, and the blue shift of characteristic adsorption peak of C–H stretching vibration occurred [30]. In addition, the spectrum of Ag nanoparticles also showed the absorption band at 1560 cm^−1^ assigned to –COO^–^ stretching vibrations, which proved that the oleic acid and n-propylamine reacted and coated on the surface of the Ag nanoparticles. These results indicated that the surface of the Ag nanoparticles was a layer of surface-active agent which was composed of oleic acid and n-propylamine. Finally, the thermal analysis curves of Ag nanoparticles are shown in Figure 3b. Only a one-step mass loss process was observed in the temperature range from 200–400 °C, corresponding to the decomposition of the monolayer complex reacted by oleic acid and the n-propylamine, revealing the monolayer surfactant-capped structure of the Ag nanoparticles. In addition, the content of the monolayer surfactant on the surface of Ag nanoparticles was 19.6%.

### 3.2. Microstructure and Morphology of POM/Ag Nanocomposites

**Figure 4:**
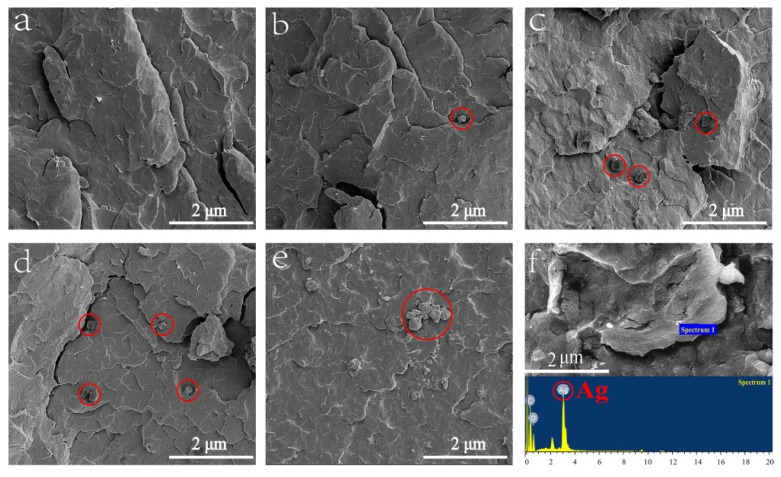
SEM images of polyoxymethylene (POM)/Ag nanocomposites with different Ag nanoparticle contents: (**a**) 0, (**b**) 0.1, (**c**) 0.5, (**d**) 1 and (**e**) 2 wt % (The red circles indicate Ag nanoparticles). (**f**) EDS results obtained at region.

SEM images of cross-section of POM/Ag nanocomposites are shown in Figure 4. Figure 4a shows the SEM image of pure POM; it can be observed that there were no nanoparticles in the POM matrix. However, it was observed that the Ag nanoparticles exhibited an average particle size of 200 nm dispersed in the POM matrix, as shown in Figure 4b–d. In addition, when the content of Ag nanoparticles was less than 1 wt %, Ag nanoparticles were well dispersed in the POM matrix, which was due to the stabilization action of the monolayer surfactants on the surface of the Ag nanoparticles. Nevertheless, when the content of Ag nanoparticles reached 2 wt % (Figure 4e), Ag nanoparticles would be easily aggregated together, which resulted in poor dispersion of Ag nanoparticles [31]. Furthermore, the Ag nanoparticles were characterized by EDS (Figure 4f). This proved that the chemical composition of Ag nanoparticles consisted of Ag atoms.

### 3.3. Melting and Non-Isothermal Crystallization Behavior of POM/Ag Nanocomposites

**Figure 5:**
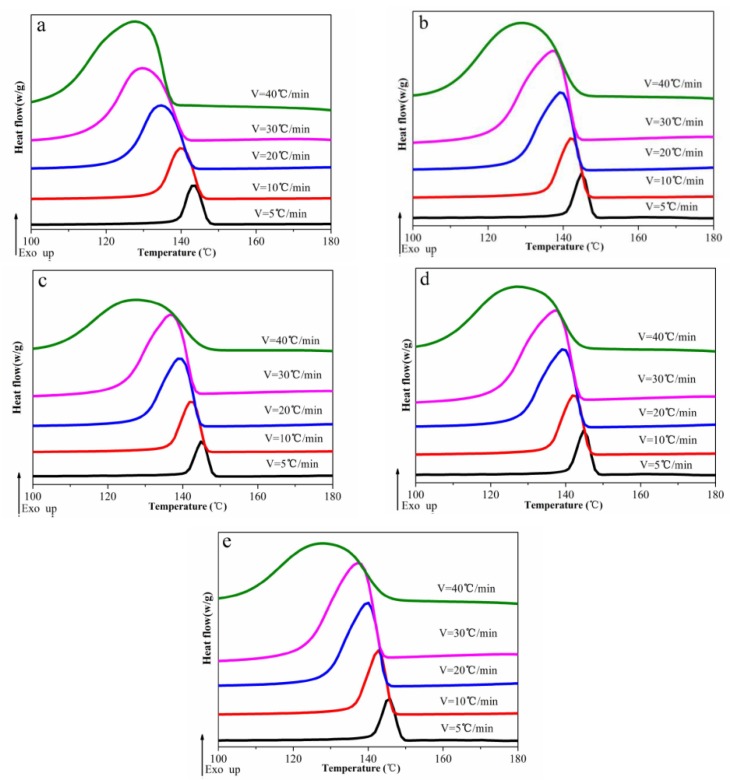
DSC cooling scan curves of POM/Ag nanocomposites with different Ag nanoparticle contents: (**a**) 0, (**b**) 0.1, (**c**) 0.5, (**d**) 1, (**e**) 2 wt % at the cooling rates of 5, 10, 20, 30 and 40 °C/min.

The non-isothermal crystallization behavior of the POM/Ag nanocomposites was studied and shown in Figure 5. The crystallization peak temperature (*T_P_*) and relative degree of crystallinity (*X_C_*) were adopted to describe the non-isothermal crystallization behavior of POM/Ag nanocomposites (Table 1 and Table 2). It was found that the *T_P_* shifted to lower temperature with the increase of the cooling rate. This may be attributed to that when the cooling rates increased, the time of the motion and rearrangement of POM macromolecular chains decreased, which made the formation of nuclei difficult. More interestingly, it was found that the *T_P_* and *X_C_* of POM/Ag nanocomposites were higher than that of the POM at the same cooling rate. This could be attributed to the fact that the efficient heterogeneous nucleation of Ag nanoparticles could enhance the crystallization rate of POM and increase the crystallinity of POM to a higher level. As the amount of Ag nanoparticles increased, the *T_P_* of the POM/Ag nanocomposites increased slightly [32]. However, when the amount of Ag nanoparticles was 2 wt%, the *X_C_* of the POM/Ag nanocomposites was lower than that of the POM/Ag nanocomposites with 1 wt% Ag nanoparticles, indicating that a high loading of Ag nanoparticles in POM/Ag nanocomposites could inhibit their crystallization to some extent [33]. This is because more Ag nanoparticles would be easily aggregated together in the POM matrix, which could affect the heterogeneous nucleation of Ag nanoparticles and crystallization performances of the POM. This was in agreement with the results from the SEM analysis.

### 3.4. Kinetics of Non-Isothermal Crystallization

#### 3.4.1. Jeziorny Model Analysis

The calculation of the relative degree of crystallinity (*X*(*T*)) is displayed in Equation (3) [34]; the relationship between time (*t*) and temperature (*T*) was as given in Equation (4):

(3) $$X\left(T\right)=\frac{\int_{T_{0}}^{T}\left(dH_{c}/dT\right)dT}{\Delta H_{C}}$$

(4) $$t=\frac{T_{0}-T}{\Phi }$$

where *T*_0_ and *T* correspond to the onset and an arbitrary temperature, respectively. d*H_C_* is the enthalpy of crystallization released during an infinitesimal temperature range d*T*, ∆*H_C_* is the overall enthalpy of crystallization for a certain cooling rate, and *Φ* is the cooling rate.

**Figure 6:**
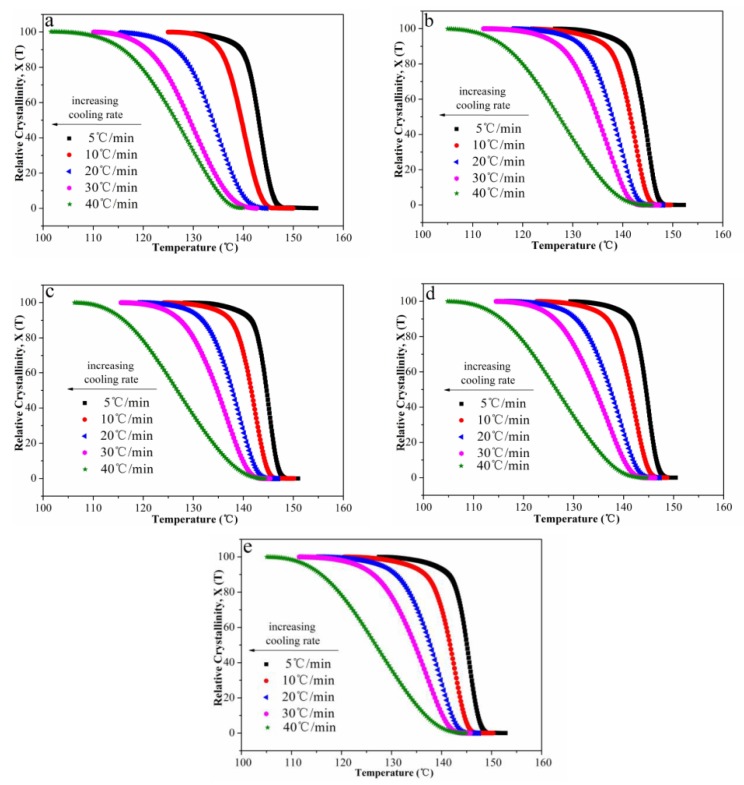
Relative degree of crystallinity as a function of temperature for POM/Ag nanocomposites with different Ag nanoparticle contents: (**a**) 0, (**b**) 0.1, (**c**) 0.5, (**d**) 1, (**e**) 2 wt % at the cooling rates of 5, 10, 20, 30 and 40 °C/min.

**Figure 7:**
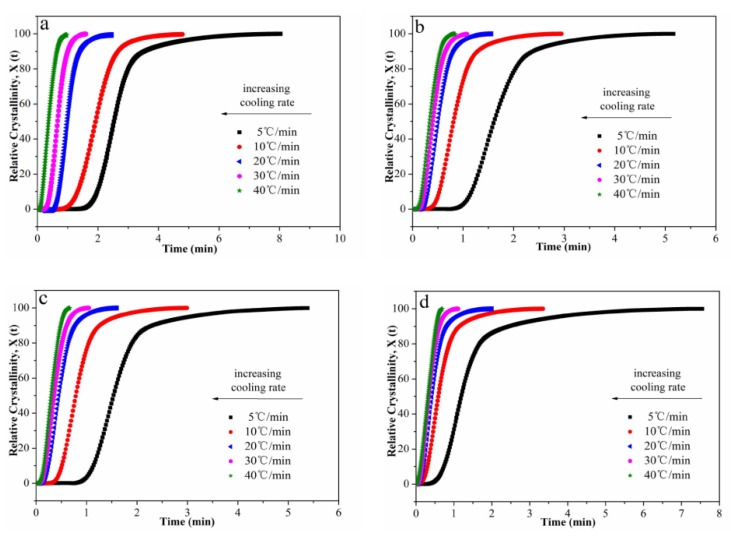

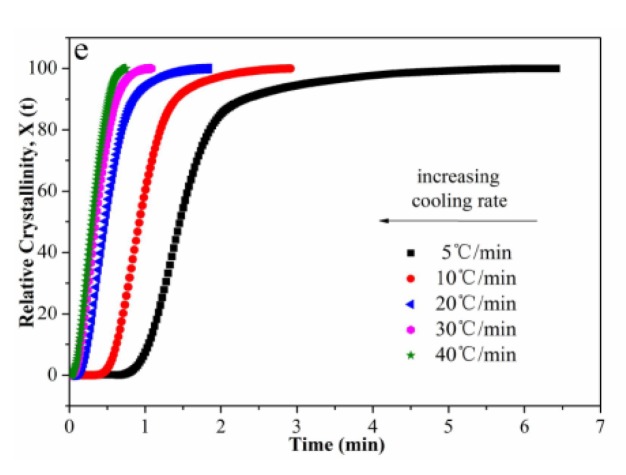
Relative degree of crystallinity as a function of time for POM/Ag nanocomposites with different Ag nanoparticle contents: (**a**) 0, (**b**) 0.1, (**c**) 0.5, (**d**) 1, (**e**) 2 wt % at the cooling rates of 5, 10, 20, 30 and 40 °C/min.

The relative degree of crystallinity (*X*(*T*)) with temperature and time are shown in Figure 6 and Figure 7, respectively. In addition, the half-time of crystallization (*t*_1/2_) values are given in Table 2. On one hand, the *t*_1/2_ values decreased with the increasing cooling rate, indicating that all samples crystallized faster when the cooling rate increased. On the other hand, in a fixed cooling rate, as the content of Ag nanoparticles increased, the *t*_1/2_ values decreased first, then increased. The *t*_1/2_ value of POM/Ag nanocomposites with 1 wt% Ag nanoparticles was smallest. It was demonstrated that when the content of Ag nanoparticles was less than 1 wt%, the Ag nanoparticles enhanced the crystallization rate of POM due to their nucleation effect. However, when the content of Ag nanoparticles was reached 2 wt%, Ag nanoparticles easily aggregated together, which had a certain inhibitory action on POM crystallization.

**Figure 8:**
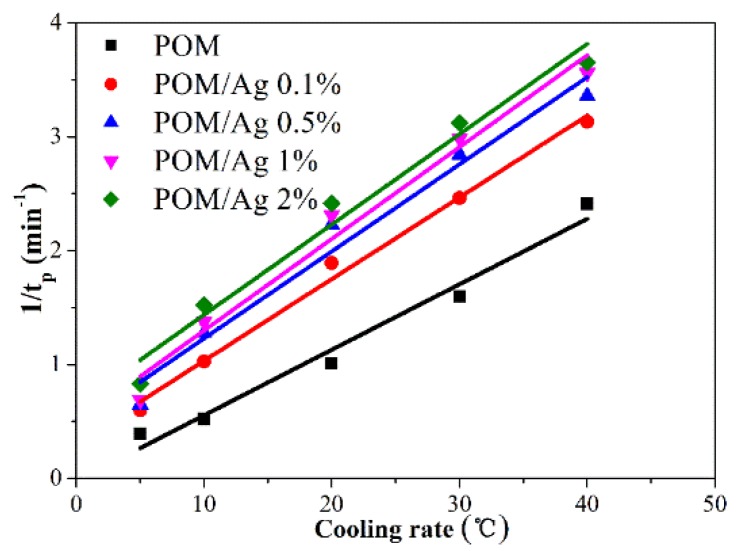
Reciprocal half-time of crystallization versus cooling rate plot of POM and POM/Ag nanocomposites.

The crystallization rate parameter (CRP) was employed to quantitatively compare the non-isothermal crystallization rates of POM/Ag nanocomposites, which could be evaluated from the function of 1/*t*_1/2_ and *Φ* [35]. The plot of 1/*t*_1/2_ versus *Φ* is shown in Figure 8, and the CPR values are given in Table 2. The CRP values of POM/Ag nanocomposites with 0, 0.1, 0.5, 1 and 2 wt % Ag nanoparticles were 0.0575, 0.0717, 0.0764, 0.0805 and 0.0792, respectively. These CRP values clearly suggest that the crystallization rate of POM was enhanced by the Ag nanoparticles when the amount of Ag nanoparticles was less than 1 wt %, due to their nucleation effect. However, when the content of Ag nanoparticles was high, they had a certain inhibitory effect on the crystallization of POM.

#### 3.4.2. Jeziorny-Modified Avrami Model Analysis

**Figure 9:**
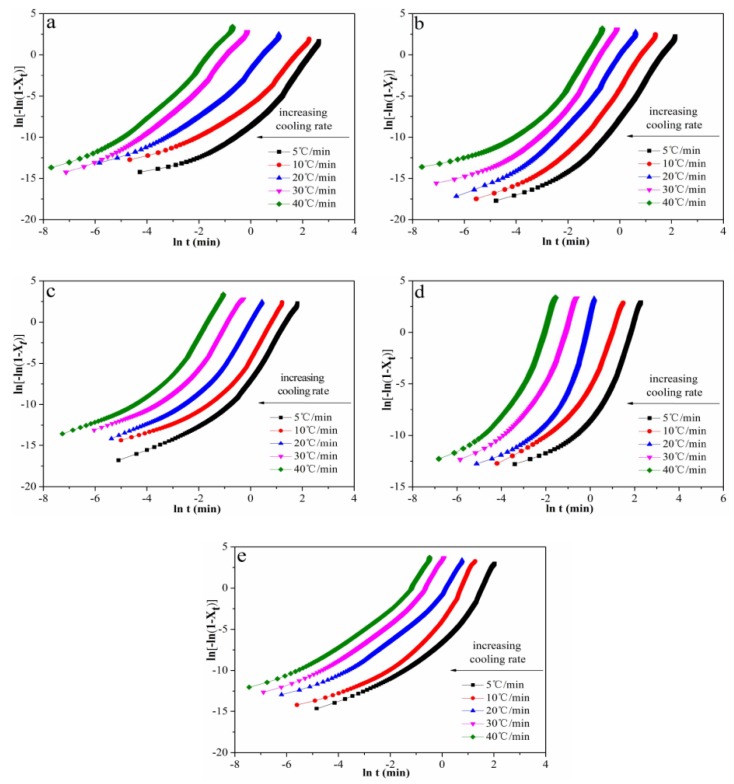
Plots of ln(−ln(1 − *X_t_*)) as a function of ln(*t*) for POM/Ag nanocomposites with different Ag nanoparticle contents: (**a**) 0, (**b**) 0.1, (**c**) 0.5, (**d**) 1, (**e**) 2 wt % at the cooling rates of 5, 10, 20, 30 and 40 °C/min.

The Jeziorny-modified Avrami model was used to describe the non-isothermal crystallization process of POM/Ag nanocomposites. In the Jeziorny-modified Avrami model [36], Equation (5) was taken in the double-logarithmic form to obtain Equation (6):

(5) $$X_{t}=1-\exp(-K_{t}t^{n})$$

(6) $$\ln[-\ln(1-X_{t})]=\ln(K_{t})+n\ln(t)$$

(7) $$\ln(K_{c})=\ln(K_{t})/\Phi$$

where *X_t_* is the relative degree of crystallinity as a function of time and *n* is the Avrami exponent, reflecting the mechanism for the nucleation and crystal growth during crystallization of polymers. *K_t_* is the crystallization rate constant. In order to eliminate the effect of cooling rate, ln(*K_t_*) was normalized with *Φ* to obtain ln(*K_C_*). Therefore, plots of ln(−ln(1 − *X_t_*)) versus ln(*t*) are shown in Figure 9, and ln(*K_C_*) values are given in Table 2. It was clearly shown that as the content of Ag nanoparticles increased, the ln(*K_C_*) values increased first, then decreased. The ln(*K_C_*) value of POM/Ag nanocomposites with 1 wt % Ag nanoparticles was the greatest. This indicated that the smaller amounts of Ag nanoparticles, i.e., less than 1 wt %, increased the crystallization rate of POM. It was clearly evident that the small amounts of Ag nanoparticles acted as the heterogeneous nuclei and raised the nucleation rate. However, when the amount of Ag nanoparticles was increased and reached 2 wt %, the Ag nanoparticles aggregated in the POM matrix and restrained crystallization of POM to some degree. In addition, the *n* value of POM was less than that of the POM/Ag nanocomposites, proving that Ag nanoparticles acted as heterogeneous nucleating agents and increased the crystal nuclei of POM.

#### 3.4.3. Ozawa Model Analysis

Ozawa extended the Avrami equation for non-isothermal crystallization analysis [37]. The relative crystallinity (*X_T_*) can be represented as a function of cooling rate, as in Equation (8):

(8) $$1-X_{T}=\exp(-K(T)/\Phi^{m})$$

(9) $$\ln[-\ln(1-X_{T})]=\ln K(T)-m\ln\Phi$$

where *m* is the Ozawa exponent. *K*(*T*) is the Ozawa crystallization rate function.

**Figure 10:**
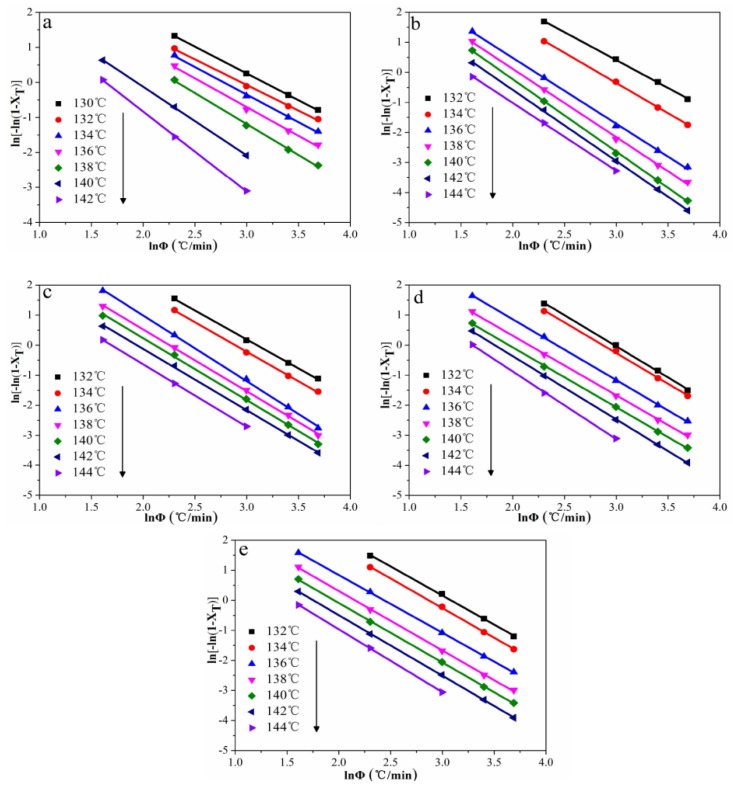
Plots of ln(−ln(1 − *X_T_*)) as a function of ln*Φ* for POM/Ag nanocomposites with different Ag nanoparticle contents: (**a**) 0, (**b**) 0.1, (**c**) 0.5, (**d**) 1, (**e**) 2 wt % at different fixed temperature.

Plots of ln(−ln(1 − *X_T_*)) versus ln(*Φ*) are shown in Figure 10. The values of *m* and ln*K*(*T*) could be calculated from the slopes and intercepts of the fitted lines, as listed in Table 3. The results indicate that the ln*K*(*T*) values of POM/Ag nanocomposites decreased when the temperature increased from low temperature to high temperature. This suggested that the crystallization rate of POM/Ag nanocomposites decreased with increasing temperature. Furthermore, the ln*K*(*T*) values of POM/Ag nanocomposites was greater than that of POM, which indicated that Ag nanoparticles enhanced the crystallization rate of POM.

#### 3.4.4. Liu and Mo’s Model Analysis

Liu and Mo’s model [38], which is a combination of Avarima and Ozawa models, is given as Equation (10):

(10) lnΦ=lnF(T)−αlnt

where *α* is the ratio of Avrami exponent to Ozawa exponent: *α* = (*m*/*n*). *F*(*T*) = (*K*(*T*)/*Z_t_*)^1/*m*^, referring to a value of cooling rate which has to be chosen at a unit crystallization time when the samples reach a certain degree of crystallinity. At a fixed relative degree of crystallinity, *F*(*T*) and *α* were calculated from the intercept and slope of the linear relationship between ln(*t*) and ln(*Φ*), respectively (Table 4). The *F*(*T*) values of POM/Ag nanocomposites were found to increase steadily with the increase in the relative degree of crystallinity, which indicated that a higher degree of relative degree of crystallinity could be obtained with a higher cooling rate. Furthermore, at a selected degree of crystallinity, it was clearly shown that as the content of Ag nanoparticles increased, the *F*(*T*) value decreased first, then increased, and the *F*(*T*) value of POM/Ag nanocomposites with 1 wt% Ag nanoparticles was least. This result was in good accordance with the results from analysis by Jeziorny, Jeziorny-modified Avrami and Ozawa models.

#### 3.4.5. Ziabicki Model Analysis

**Figure 11:**
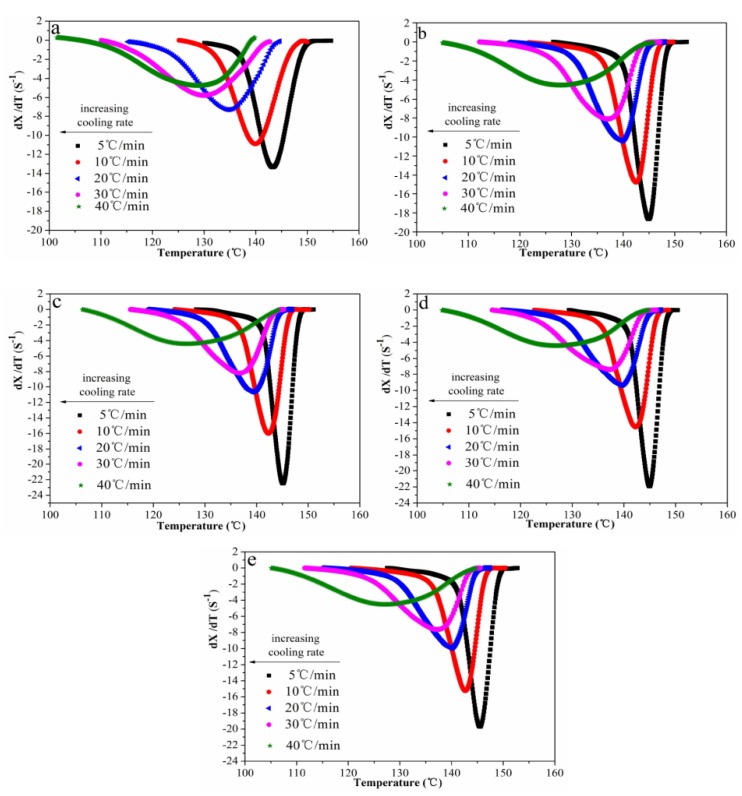
Crystallization rate (d*X*/d*T*) as a function of temperature for POM/Ag nanocomposites with different Ag nanoparticle contents: (**a**) 0, (**b**) 0.1, (**c**) 0.5, (**d**) 1, (**e**) 2 wt % at the cooling rates of 5, 10, 20, 30 and 40 °C/min.

Ziabicki proposed a model to analyze non-isothermal crystallization kinetics of POM/Ag nanocomposites. Termed Ziabicki kinetics, it is given as Equation (11) [39]:

(11) $$G_{Z,\Phi }=\int_{T_{g}}^{T_{m}^{0}}{\left(dX/dT\right)}_{\Phi }dT\approx 1.064(dX/dT{)}_{\Phi ,\max}D_{\Phi }$$

where (d*X*/d*T*)*_Φ_*_,max_ and *D_Φ_* are the maximum crystallization rate and the half-width of the derivative of relative crystallinity, respectively. $T_{m}^{o}$ is the equilibrium melting temperature. *T_g_* is the glass transition temperature. Plots of (d*X*/d*T*) versus *T* are shown in Figure 11. The value of kinetic crystallizability at unit cooling rate (*G_Z_*) can be obtained by normalizing *G_Z,Φ_* with *Φ* (Table 5). It was found that as the content of Ag nanoparticles increased, the *F*(*T*) value increased first, then decreased, and the *G_Z_* value of POM/Ag nanocomposites with 1 wt % Ag nanoparticles was greatest. This showed that crystallization rate of POM was enhanced by the small amount of Ag nanoparticles due to their nucleation effect. However, when the content of Ag nanoparticles reached 2 wt %, Ag nanoparticles easily aggregated, which had a certain inhibitory action on POM crystallization.

#### 3.4.6. Kissinger Model Analysis

**Figure 12:**
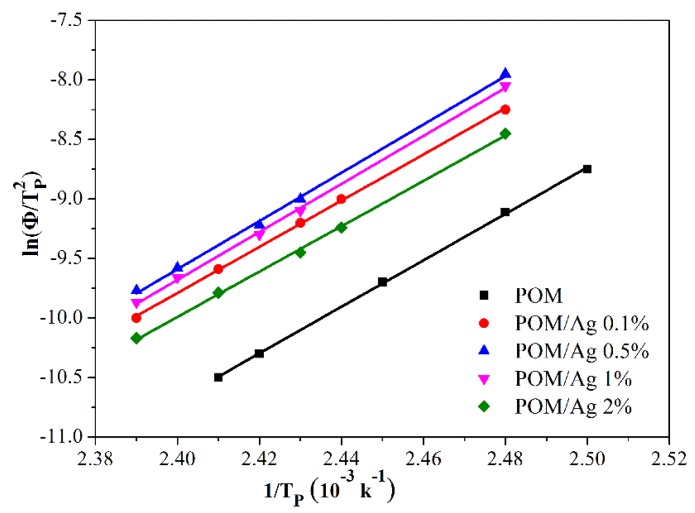
Plots of ln(*Φ*/$T_{P}^{2}$) versus 1/*T_P_* for the POM/Ag nanocomposites.

In order to calculate the activation energy of the POM/Ag nanocomposites’ crystallization, Kissinger provided an equation, given as Equation (12) [40]:

(12) $$\frac{d\left(\ln\left(\Phi /T_{P}^{2}\right)\right)}{d\left(1/T_{P}\right)}=-\frac{\Delta E}{R}$$

where ∆*E* and R are the crystallization activation energy and the gas constant, respectively. The fitted lines of ln(*Φ*/$T_{p}^{2}$) versus 1/*T_p_* for POM/Ag nanocomposites are shown in Figure 12, and the values of *r* and ∆*E* are listed in Table 6. The ∆*E* values of the POM/Ag nanocomposites indicated that Ag nanoparticles induced the heterogeneous nucleation in POM by lowering the activation energy of POM. In addition, it is known that the magnitude of activation energy (|∆*E*|) is related to energy need for the motion of polymer chains during the transformation from the melt into the crystalline state. The absolute values of ∆*E* for POM/Ag nanocomposites were greater than that for the POM, indicating that Ag nanoparticles increased the difficulty for the transport of POM molecular chains to the crystal region [41]. On one hand, it was shown that the as-synthesized Ag nanoparticles had a strong interaction with POM. On the other hand, when the amount of Ag nanoparticles increased, Ag nanoparticles easily to aggregated and inhibited the movement of the POM molecular chain, which affected the crystallization properties of the POM.

### 3.5. Morphology of POM/Ag Nanocomposites Spherulites

**Figure 13:**
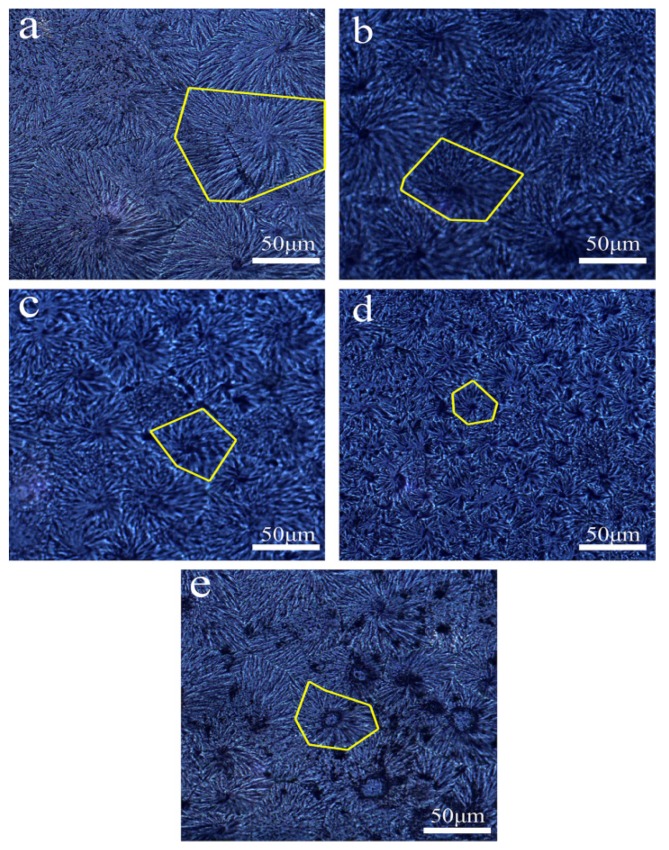
Polarized optical microscopy photographs of POM/Ag nanocomposites with different Ag nanoparticle contents: (**a**) 0, (**b**) 0.1, (**c**) 0.5, (**d**) 1, (**e**) 2 wt % (The yellow parts indicate spherulites).

The spherulite morphology of POM was observed by polarized optical microscopy to investigate the effect of the Ag nanoparticles on the dynamic crystallization behavior of POM. Figure 13 displays spherulite morphology of the POM/Ag nanocomposites. As seen in Figure 13a, the POM spherulites had considerable size due to the low nucleation density of the pure POM, and spherulites directly collided with each other. In the observed areas, there were a few nuclei and spherulites with a diameter of approximately 100 μm, along with clear interfaces. In addition, Figure 13b–e shows that the size of spherulites of POM/Ag nanocomposites became small and that the amount of the spherulites increased significantly. Furthermore, during the crystallization process, Ag nanoparticles generated a great number of nuclei and the maximum number of available nucleation sites [42]. Simultaneously, Ag nanoparticles grew in a limited space and led to the formation of smaller spherulites in the composites, indicating that the nucleation density was the dominant factor affecting the crystallization process of the POM/Ag nanocomposites [43]. However, when the content of Ag nanoparticles was increased further to reach 2 wt%, Ag nanoparticles easily aggregated, which reduced heterogeneous nucleation of Ag nanoparticles and restrained crystallization of POM.

### 3.6. Antibacterial Activities of POM/Ag Nanocomposites

**Figure 14:**
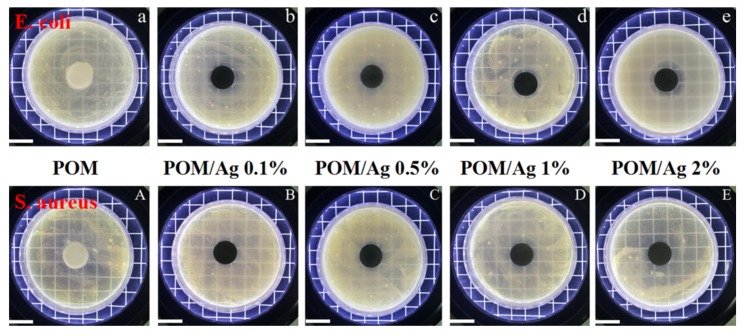
Optical images of the inhibition zones of POM/Ag nanocomposites against *E. coli* (**a**–**e**) and *S. aureus* (**A**–**E**). The inset scale bar is 2 cm.

The optical images of inhibition zones of POM/Ag nanocomposites are displayed in Figure 14, and the inhibition zones and inhibition rates of POM/Ag nanocomposites are given in Table 7. Notably, POM did not present any antibacterial activity under the test condition, as shown in Figure 14a,A. Comparatively, it can be seen in Table 7 that POM/Ag nanocomposites with 0.1 wt % Ag nanoparticles showed very promising antibacterial performance and that the inhibition zones were 0.31 cm against *E. coli* and 0.30 cm against *S. aureus*. The inhibition zones of POM/Ag nanocomposites against *E. coli* widened from 0.31 to 0.57 cm, and those against *S. aureus* widened from 0.30 to 0.53 cm when the addition of Ag nanoparticles increased from 0.1 wt % to 2 wt %. In addition, when the content of Ag nanoparticles was 0.1 wt %, the inhibition rates of POM/Ag nanocomposites against *E. coli* and *S. aureus* were 87.23% and 86.59%, respectively. The inhibition rates of POM/Ag nanocomposites against *E. coli* and *S. aureus* were both found to increase with the increasing content of Ag nanoparticles. Furthermore, when the content of Ag nanoparticles reached 2 wt %, inhibition rates of POM/Ag nanocomposites against *E. coli* and *S. aureus* were 98.12% and 97.67%, respectively. The release of Ag atoms or ions from Ag nanoparticles had been considered as one major route to produce bactericidal effects [44]. Ag ions can interact with the cell membranes, nucleic acids and proteins of the bacteria via ligand exchange reactions to exert their activity [26]. Additionally, the direct interaction of Ag nanoparticles with the surface of bacterial cells also plays an important role in their antibacterial activity [45].

## 4. Conclusions

Ag nanoparticles were synthesized by a facile route in the presence of oleic acid and n-propylamine. The crystal structure of as-synthesized Ag nanoparticles was studied by XRD, and the average primary size of the Ag nanoparticles was approximately 10 nm. The surface of Ag nanoparticles was capped with monolayer surfactants which consisted of n-propylamine and oleic acid with the content of 19.6%, which was proved by FTIR and TGA. Based on as-synthesized Ag nanoparticles, POM/Ag nanocomposites were prepared by a simple melt compounding route. It was found that when the content of Ag nanoparticles in the POM matrix was less than 1 wt %, the Ag nanoparticles showed good dispersibility in the POM. Furthermore, the effect of Ag nanoparticles on the crystallization behavior and non-isothermal kinetics of POM was investigated by DSC. Addition of Ag nanoparticles dramatically enhanced the crystallization peak temperature (*T_P_*) and relative degree of crystallinity (*X_C_*) of POM. All of the Jeziorny, Jeziorny-modified Avrami, Ozawa, Liu and Mo’s analysis models were found to describe the non-isothermal kinetics of samples fairly well. The results showed that well-dispersed Ag nanoparticles in POM matrix could decrease its half-time of crystallization (*t*_1/2_) and increase the crystallization rate parameter (CRP) values of POM. The values of the crystallization rate constant at unit cooling rate (ln(*K_C_*)) and the crystallization rate function (*K*(*T*)) for POM both were less than that for POM/Ag nanocomposites, and the values of ln(*K_C_*) and *K*(*T*) of POM/Ag nanocomposites with 1 wt % Ag nanoparticles was the greatest. Furthermore, to demonstrate the characterization of the kinetic crystallinity of the samples for a certain cooling rate, the values of kinetic crystallizability at unit cooling rate (*Gz*) of the Ziabicki model analysis were found to be in the following order: *Gz* (POM/Ag nanocomposites) > *Gz* (POM). Similarly, Kissinger model analysis revealed that the crystallization activation energy (∆*E*) value of POM was −167.70 kJ/mol, greater than that of POM/Ag nanocomposites. Furthermore, all results regarding the kinetics of non-isothermal crystallization explained that when the amount of Ag nanoparticles was less than 1 wt %, Ag nanoparticles acted as heterogeneous nuclei of POM and generated a great number of nuclei, which reduced the spherulites size of POM and increased the number of POM spherulites, while increasing the crystallization rate of POM. However, when the amount of Ag nanoparticles reached 2 wt %, the Ag nanoparticles in POM easily to aggregated and inhibited the movement of the POM molecular chain, which reduced the effect of heterogeneous nucleation of Ag nanoparticles in POM. In addition, the POM/Ag nanocomposites showed robust antibacterial activity against *E. coli* and *S. aureus*, compared with that of POM. When the content of Ag nanoparticles was reached 2 wt %, inhibition rates of POM/Ag nanocomposites were greater than 97%.

## Figures and Tables

**Table 1:** Characteristic crystallization peak temperatures. Enthalpy and degree of crystallinity values of samples crystallized with the cooling rate of 10 °C/min.

Samples	*T_c,o_* (°C)	*T_c,p_* (°C)	∆*H_m_* (J/g) ^a^	*X_C_* (%) ^b^
POM	146.12	139.64	152.94	46.91
POM/Ag 0.1%	147.85	141.83	157.89	48.43
POM/Ag 0.5%	148.21	142.81	164.08	50.33
POM/Ag 1%	148.96	142.83	166.64	51.12
POM/Ag 2%	148.33	141.71	163.71	50.21

^a^ Enthalpy of second melting endotherm recorded at the heating rate of 10 °C/min. ^b^ Degree of crystallinity calculated with Equation (1) by using the enthalpy values of second melting.

**Table 2:** Parameters of Jeziorny and Jeziorny-modified Avrami analysis for the POM/Ag nanocomposites.

Samples	*Φ* (°C/min)	*T_P_* (°C)	*t*_½_ (min)	*r*	CPR	*n*	ln(*K_t_*)	ln(*K_C_*)
POM	5	141.82	2.53	0.947	0.0575	3.04	−8.107	−1.621
	10	139.64	2.13	0.956		3.23	−5.129	−0.513
	20	132.77	0.84	0.931		3.03	−3.252	−0.163
	30	129.57	0.58	0.953		3.01	−1.043	−0.035
	40	127.88	0.33	0.981		3.19	1.912	0.049
POM/Ag 0.1%	5	144.83	1.61	0.975	0.0717	3.92	−5.021	−1.004
	10	141.83	0.81	0.966		3.94	−1.530	−0.153
	20	139.03	0.50	0.972		3.95	0.027	0.001
	30	137.90	0.39	0.971		3.96	1.847	0.061
	40	129.98	0.33	0.970		3.91	2.323	0.058
POM/Ag 0.5%	5	144.84	1.53	0.975	0.0764	3.98	−4.792	−0.958
	10	142.81	0.78	0.975		3.94	−2.671	−0.267
	20	139.71	0.44	0.988		3.95	−0.125	−0.006
	30	137.74	0.35	0.976		3.93	1.005	0.034
	40	129.89	0.29	0.967		3.91	2.158	0.054
POM/Ag 1%	5	144.83	1.26	0.958	0.0805	3.99	−4.763	−0.953
	10	142.83	0.71	0.968		3.92	−2.494	−0.249
	20	140.03	0.40	0.981		4.00	0.349	0.017
	30	137.90	0.32	0.972		3.92	1.632	0.054
	40	129.98	0.29	0.974		3.98	2.795	0.069
POM/Ag 2%	5	143.83	1.39	0.977	0.0792	3.68	−5.641	−1.128
	10	141.71	0.78	0.981		3.88	−3.086	−0.308
	20	138.84	0.41	0.986		3.71	−0.679	−0.034
	30	137.54	0.33	0.991		3.90	1.151	0.038
	40	127.54	0.31	0.984		3.85	2.147	0.054

**Table 3:** Parameters of Ozawa analysis for the POM/Ag nanocomposites.

Samples	*T* (°C)	*r*	*m*	ln*K*(*T*)	Samples	*T* (°C)	*r*	*m*	ln*K*(*T*)
POM	130	0.999	1.52	4.84	POM/Ag 0.1%	132	0.999	2.01	5.97
	132	0.997	1.46	4.31		134	0.999	2.10	5.68
	134	0.997	1.57	4.36		136	0.999	2.20	4.80
	136	0.999	1.64	4.21		138	0.998	2.19	4.63
	138	0.999	1.77	4.11		140	0.999	2.31	4.40
	140	0.999	1.96	3.80		142	0.999	2.37	4.10
	142	0.999	2.20	3.75		144	0.999	2.17	3.87
POM/Ag 0.5%	132	0.999	1.99	5.97	POM/Ag 1%	132	0.999	2.07	6.17
	134	0.999	2.01	5.69		134	0.997	2.04	5.85
	136	0.999	2.18	5.36		136	0.997	2.02	5.30
	138	0.999	2.05	4.64		138	0.999	2.01	4.91
	140	0.999	2.06	4.35		140	0.999	2.03	4.36
	142	0.998	2.04	4.10		142	0.999	2.10	4.12
	144	0.999	2.07	3.90		144	0.999	2.26	3.91
POM/Ag 2%	132	0.999	1.93	5.96					
	134	0.997	1.97	5.65					
	136	0.997	1.92	4.90					
	138	0.999	1.97	4.48					
	140	0.999	1.98	4.27					
	142	0.999	2.01	4.01					
	144	0.999	2.05	3.89					

**Table 4:** Crystallization kinetic parameters of the POM/Ag nanocomposites at different degrees of relative crystallinity, determined from Liu and Mo’s model.

Samples	*X_t_*	*α*	*F*(*T*)
POM	20	0.814	12.67
	40	0.873	12.94
	60	0.936	13.07
	80	1.035	13.87
POM/Ag 0.1%	20	0.714	5.291
	40	0.761	5.457
	60	0.865	5.529
	80	0.945	5.737
POM/Ag 0.5%	20	0.697	4.482
	40	0.724	4.730
	60	0.762	5.160
	80	0.823	5.818
POM/Ag 1%	20	0.713	2.664
	40	0.727	2.933
	60	0.735	3.232
	80	0.781	4.301
POM/Ag 2%	20	0.701	5.387
	40	0.759	5.801
	60	0.898	6.080
	80	0.987	6.129

**Table 5:** Parameters of Ziabicki analysis for the POM/Ag nanocomposites.

Samples	*Φ* (°C/min)	*T_Φ_*_,max_ (°C)	(d*X*/d*T*)*_Φ_*_,max_ (s^−1^)	*D_Φ_* (°C)	*G_Z,Φ_*	*G_Z_*
POM	5	143.36	13.32	6.58	93.25	18.65
	10	139.83	10.87	7.64	88.36	8.84
	20	134.96	7.29	10.98	85.17	4.26
	30	129.71	5.76	15.27	93.58	3.12
	40	128.53	4.73	16.22	81.63	2.04
Average						7.382
POM/Ag 0.1%	5	144.93	18.64	5.01	99.36	19.87
	10	142.59	14.73	6.30	98.74	9.87
	20	139.73	10.39	8.89	98.28	4.91
	30	137.04	8.11	11.42	98.54	3.28
	40	127.50	4.51	20.81	99.85	2.49
Average						8.084
POM/Ag 0.5%	5	145.14	22.44	4.16	99.32	19.86
	10	142.12	15.91	6.01	101.74	10.17
	20	139.46	10.60	8.60	96.99	4.85
	30	136.48	8.20	11.17	97.46	3.25
	40	136.17	4.41	21.94	102.95	2.57
Average						8.140
POM/Ag 1%	5	145.03	21.91	4.66	108.64	21.73
	10	142.29	14.53	6.91	106.82	10.68
	20	139.75	9.41	10.62	106.33	5.32
	30	137.42	7.35	13.27	103.78	3.46
	40	126.66	4.43	21.98	103.60	2.59
Average						8.756
POM/Ag 2%	5	145.47	19.69	4.67	97.84	19.57
	10	142.53	14.98	5.99	95.47	9.55
	20	139.20	9.72	10.01	103.52	5.18
	30	136.99	7.61	12.81	103.72	3.46
	40	124.75	4.39	21.29	99.44	2.49
Average						8.050

**Table 6:** Non-isothermal crystallization activation energy for the POM/Ag nanocomposites calculated using the Kissinger model.

Samples	POM	POM/Ag 0.1%	POM/Ag 0.5%	POM/Ag 1%	POM/Ag 2%
Activation energy ∆*E* (kJ/mol)	−167.70	−171.26	−171.76	−172.24	−172.38
*r*	0.998	0.999	0.997	0.998	0.997

**Table 7:** The inhibition zones and inhibition rates of POM/Ag nanocomposites.

	Samples	POM	POM/Ag 0.1%	POM/Ag 0.5%	POM/Ag 1%	POM/Ag 2%
*E. coli*	inhibition zones (cm)	0	0.31	0.40	0.46	0.57
	Inhibition rates (%)	0	87.23	92.56	95.98	98.12
*S. aureus*	inhibition zones (cm)	0	0.30	0.38	0.43	0.53
	Inhibition rates (%)	0	86.59	91.83	94.74	97.67
